# Rheumatism

**Published:** 1887-06-25

**Authors:** 


### The Doctor's Pulpit.

### RHEUMATISM.

When the Romans occupied Britain they had fortified camps in various parts of the country, from which they could sally forth on the slightest symptom of an outbreak of rebellion, and nip the dangerous rising in the bud. The man who, having a tendency to rheumatism, wishes to preserve himself from dangerous attacks, should regard his body as a camp to be kept well fortified by vigorous general health, and on the slightest symptom of the breakdown of that general health should take immediate steps for its repair and complete restoration. For if a man's house be his castle, his body and mind are his kingdom, which he holds in fee simple from his Creator. He has, or should have, complete authority in his own kingdom, and that he is held to a strict accountability for the use he makes of it the evidence of universal experience goes to show. Some people think that only deeds which are wilfully bad should be punished. Nature does not agree with them. She punishes with great rigour, not only bad people, but stupid people, ignorant people, idle people, and generally all people who either have not the sense or have not the will to understand and obey her teachings. A man may be a decidedly moral and religious person, and at the same time a very great fool. Nature does not provide against his folly because he is religious. She simply allows him to take his chance like other people. Many a preacher and missionary and district visitor thinks that because he is engaged in a good work there will be special provision made to prevent his breaking down. Experience is dead against any such view of things. Not only so, but there is no encouragement in the Christian Scriptures for any theory of the kind. "As a man sows so will he reap," is the dictum of the Old and New Testaments, and modern science confirms to the very letter so sound and eminently rational a principle.

All this seems like wandering away from the text; but it is not so. There are few or no tendencies so decidedly under the control of man as the tendency to rheumatism. Many people are born with a rheumatoid constitution. When we see symptoms of rheumatic fever or pains in a person, be they in man, woman, or child, one of the first questions we ask is, "Did your parents or grandparents suffer from rheumatism; or do you remember anything of it in uncles, aunts, or cousins?" For rheumatism is emphatically one of the hereditary "diatheses," as they are called, *i.e.*, the tendency to it is transmitted from parent to offspring.

On this account, if young people ever took advice, we would advise rheumatic men to avoid marrying rheumatic women. And similarly we would say to rheumatic young women, "If any pale-faced, fair-haired, large-tonsilled, jointy young man should show symptoms of falling in love with you, do not on any account encourage him. Rather by half take a dark-skinned, black-haired, biliously inclined Benedict; or, still better, a tall, strapping fellow of sanguine disposition, even if he have rather an empty head. Boys and men, and women and girls too, who have the rheumatic diathesis soon become recognised by observant doctors. Whilst, therefore, we cannot expect young people to have so much medico-scientific knowledge as to distinguish the rheumatic youth from others, we may suggest that their intelligent family doctor should be consulted if there be any suspicion of hereditary taint, and his advice given due heed to. This is not said in the interests of doctors—far from it. For if there be one kind of case more than another which the sensible doctor does not like to be consulted about, it is where Jemima seeks advice about George, or George takes the doctor's opinion of Jemima. Because all the while Jemima knows that if George had a wooden leg as well as a rheumatic constitution, she would marry no one but him. "Dear George—to think I should throw you over, because that horrid doctor said you might have rheumatic fever and heart-disease some day.—I hate doctors!" And George himself, like the true-hearted fellow he is, fully reciprocates the sentiment. And we do not blame him when things have really gone so far as this. We simply venture to suggest that at the very outset, the extremest margin of love-making, it might be worth while to consider whether the dark-haired Robert or the brown-bearded Dick would not really suit you quite as well, if you yourself have already a tendency to rheumatism. Of course, we do not think of insisting even in such a case as this. We merely offer a scientific strawberry for the consideration of your nineteenth century palate.

Twenty years ago, when a man had acute rheumatism, as rheumatic fever is called, the principal medicaments were "patience and flannel." The doctor prescribed the former, but he did not say where it was to be dispensed, whether at the family chemist's, or the "Cheap and Nasty" Stores. Nowadays, salicin in some form is used. It is an active principle obtained from the willow plant, and was introduced to the notice of the profession by Dr. Maclagan, then of Dundee, now of London. Dr. Maclagan rode into fame on the back of a willow stick: as good a broom-handle for a riding-horse as many another. The new drug had very properly to run the gauntlet of the hospitals; and after its fiery trial it came out abundantly triumphant. It is emphatically a drug which, if it could not be purchased cheaper, is worth a guinea an ounce. But let no wrong-headed person run away with the idea that he can cure himself of rheumatic fever by dosing himself with salicin. Our advice is—never touch salicin except under the direction of an intelligent and trustworthy doctor. It is a powerful poison and a dangerous toy to play with. A further piece of advice, and quite as important, is this: on the slightest symptoms of severe rheumatism, send for the doctor immediately. It is highly probable that many cases of heart-disease, resulting from rheumatic fever, might be entirely, or almost entirely prevented by the very early and skilful use of salicin. Send, therefore, for the doctor without an hour's delay.

But undoubtedly "prevention" is the jewel of by far the greatest price. An ounce of prevention is worth a million tons of cure. The present writer would rather pay the doctor a hundred pounds for preventing an attack of rheumatic fever, than five shillings for curing it. This miserable disease is like a big bully at school: if once it gets a man well into its power, it will seldom let go its hold completely. It often leaves him seriously disabled, and very frequently returns again and again to give him a six weeks' banishment from business and all useful work. One poor man, well known to the writer, has had the disease thirteen times within twenty years. He is still alive; and, thanks to salicin, does not suffer nearly so much as formerly. But let it be repeated in the largest possible print, and stored away on the most convenient shelf in the memory, "that an ounce of prevention is worth a pound of cure," and that the best way of prevention is by moderate work, moderate food, sufficient exercise, and avoidance of worry, to maintain constantly a steady level of robust general health.

